# Community Structure and Function of Amphibian Skin Microbes: An Experiment with Bullfrogs Exposed to a Chytrid Fungus

**DOI:** 10.1371/journal.pone.0139848

**Published:** 2015-10-07

**Authors:** Jenifer B. Walke, Matthew H. Becker, Stephen C. Loftus, Leanna L. House, Thais L. Teotonio, Kevin P. C. Minbiole, Lisa K. Belden

**Affiliations:** 1 Department of Biological Sciences, Virginia Tech, Blacksburg, VA, United States of America; 2 Department of Statistics, Virginia Tech, Blacksburg, VA, United States of America; 3 Department of Chemistry, James Madison University, Harrisonburg, VA, United States of America; 4 Department of Chemistry, Villanova University, Philadelphia, PA, United States of America; Imperial College Faculty of Medicine, UNITED KINGDOM

## Abstract

The vertebrate microbiome contributes to disease resistance, but few experiments have examined the link between microbiome community structure and disease resistance functions. Chytridiomycosis, a major cause of amphibian population declines, is a skin disease caused by the fungus, *Batrachochytrium dendrobatidis* (Bd). In a factorial experiment, bullfrog skin microbiota was reduced with antibiotics, augmented with an anti-Bd bacterial isolate (*Janthinobacterium lividum)*, or unmanipulated, and individuals were then either exposed or not exposed to Bd. We found that the microbial community structure of individual frogs prior to Bd exposure influenced Bd infection intensity one week following exposure, which, in turn, was negatively correlated with proportional growth during the experiment. Microbial community structure and function differed among unmanipulated, antibiotic-treated, and augmented frogs only when frogs were exposed to Bd. Bd is a selective force on microbial community structure and function, and beneficial states of microbial community structure may serve to limit the impacts of infection.

## Introduction

The ecological debate over the relationship between community structure and function has a long history [1–3]. In general, species richness has a positive effect on most ecosystem services, such as productivity and stability, but these results are based on work in relatively few systems and with few functional endpoints [4,5]. Only recently have researchers begun to examine structure-function relationships in complex microbial communities, including host-associated symbiotic communities [6,7]. Given their short generation times, relative ease of manipulation, and advanced molecular tools, these systems may be useful for revealing general patterns and mechanisms underlying structure-function relationships [8].

**Fig 1:**
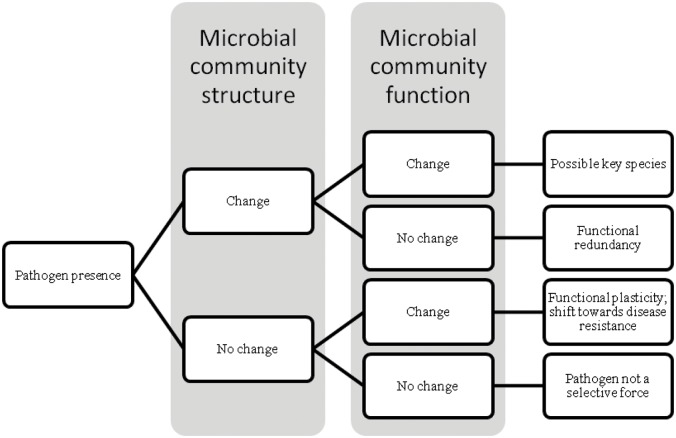
Conceptual model representing potential responses and interpretations of microbial community structure and function in the presence of a pathogen.

Here, we focus on understanding these structure-function relationships in complex bacterial symbiont communities in the context of pathogen exposure, as potentially lethal pathogens should be strong selective forces on disease resistance functions of symbiont communities (Fig 1). One potential outcome is that with pathogen presence, community structure will change as the pathogen competes with other microbes in the community. In this case, the function of the community may or may not change depending on whether bacterial symbionts are functionally redundant or there are key species present. If species are primarily redundant, the loss of one species can be offset by another, and the structure of the community may change with pathogen presence without altering the function. If, however, species make unique contributions to function, the loss or gain of a key species when a pathogen is present will alter community function. Alternatively, community structure could remain unchanged, but community function could exhibit a shift towards increased disease resistance, indicating plasticity in the functional response. Lastly, both community structure and function could remain unchanged with pathogen presence, suggesting that the pathogen is not a strong selective force on community function.

Chytridiomycosis is an amphibian skin disease caused by the fungus, *Batrachochytrium dendrobatidis* (Bd), and is a major contributor to global amphibian population declines [9]. The skin of amphibians is host to a diverse resident bacterial community, and some of these bacteria inhibit Bd [10–13]. A supplemented, protective cutaneous microbiota can reduce morbidity and mortality in amphibians infected with Bd [14], and reduction of the cutaneous microbiota can alter disease outcome [15]. The protective function of the microbiota is likely due to bacterial metabolites inhibiting zoospore colonization or development [16].

To assess the role of bacterial community structure on host health, the potential for pathogens to act as selective forces on microbiota function, and the link between the structure and disease resistance function of the skin microbiota, we manipulated (reduced or augmented) the skin microbiota of bullfrogs (*Lithobates catesbieanus*), exposed them to Bd and tracked host and skin bacterial community responses. Bullfrogs were used because they are generally not considered susceptible to chytridiomycosis ([17,18], but see [19]). Therefore, skin microbiome responses were not likely to be confounded by host mortality.

## Materials and Methods

### Experimental design

Sixty juvenile bullfrogs (6.7–13.2g, mean = 8.8g) were collected on two separate days in August 2010 from a pond in Giles County, VA (USA), where Bd has been previously detected [20,21]. In the field, collected bullfrogs had low levels of Bd infection, both in terms of prevalence (50%, 30 of the 60 frogs were infected) and intensity (mean+/-SE = 0.61+/-0.25 zoospore equivalents, range = 0–14 zoospore equivalents). Individual frogs were swabbed in the field to assess initial microbiota and Bd using a single swab. Each individual was handled with fresh gloves, rinsed twice with sterile water to remove transient microbes [22], and swabbed ten strokes along the ventral side and five strokes along each thigh and rear foot using sterile rayon swabs (MW113, Medical Wire & Equipment). We swabbed these regions because Bd infection is generally concentrated there [23]. Swabs were placed in sterile 1.5ml microcentrifuge tubes and frozen at -20C. Frogs were then returned to the laboratory and randomly assigned to one of six treatment groups in a factorial design with microbiota treatment (three levels: unmanipulated, reduced, augmented) and Bd treatment (two levels: exposed, not exposed). Frogs were housed individually at 17C on a 12h light cycle in autoclaved 15 x 33 x 23 cm containers that were slanted and contained 500ml sterile water and two sterile moist paper towels. Cages were changed and frogs were fed crickets *ad lib* twice per week throughout the experiment.

Treatments started after frogs were acclimated to the laboratory environment for one week. Frogs had their skin microbiota experimentally unmanipulated (U), reduced with antibiotics (AB), or augmented with *Janthinobacterium lividum* (JL; a bacterial culture isolated from a four-toed salamander, *Hemidactylium scutatum*, in Virginia and used in prior studies to limit Bd impacts; e.g. [14]), and were then either exposed or unexposed to Bd. We used an unbalanced design, with more frogs in the Bd exposure treatments (N = 13) than in the unexposed treatments (N = 7), because we were mainly interested in bacterial treatment effects on microbiota structure and function in the presence of Bd. To reduce the skin microbiota, frogs were housed in autoclaved containers with 500ml broad-spectrum antibiotic solution every day for 2 weeks, alternating among maracyn (5ml/L), enrofloxacin (30mg/L), and cephalexin (12mg/L), similar to Becker & Harris [15]. To augment the skin microbiota, frogs were placed in autoclaved 400ml containers with 100ml of *J*. *lividum* cells in sterile water for six hours [as in 14]. Frogs were exposed at 11 (1.8x10^8^ cells/ml) and 4 (3.8x10^7^ cells/ml) days before exposure to Bd, which occurred on experiment day 0. Frogs in the unmanipulated microbiota treatment were placed in 100ml of sterile water for the same time period to control for handling effects.

To maximize the potential impact of Bd on the skin microbiota, a Panamanian Bd strain (JEL310) was used. This strain was lethal to another North American amphibian species [24] that did not experience mortality from a North American Bd isolate [15]. While we did not want mortality to confound impacts of Bd on the microbiota, we did want to optimize the potential effects of Bd exposure on bullfrogs’ microbiota. Frogs (N = 13/microbiota manipulation) were exposed to Bd at day 0 by placing them in 100ml solutions of 7x10^6^ zoospores for 24 hours in autoclaved 400ml containers. Unexposed frogs (N = 7/pre-treatment) were placed in sterile water. To ensure successful infection, a second Bd exposure (4.8x10^7^ zoospores) was performed at day 4.

Frogs were swabbed for bacterial community analysis and Bd infection intensity initially in the field (as described above, day –21), after microbiota manipulations and prior to Bd exposure (day 0), and one week after exposure to Bd (day 7). A second swab for metabolite profiles was also taken in the same manner at days 0 and 7. Metabolite analysis was performed on day 7 samples. The same sterile rayon swabs (MW113, Medical Wire & Equipment) were used for DNA and metabolite collection. Frog mass was also recorded at days 0, 7, and 42, as mass loss is a common sublethal effect of Bd exposure (e.g. [25]). No frogs died during the experiment, which lasted 42 days.

### Microbial community analysis

Ten randomly chosen frogs of the 13 in each Bd exposed treatment and all 7 frogs in each no Bd treatment were used for microbial community analysis. DNA from bacterial swabs was extracted using the Qiagen DNeasy Blood & Tissue Kit (Valencia, CA) with lysozyme pretreatment. The V4 region of the 16S rRNA gene was amplified using the primers 515F and barcoded 806R [26,27]. For each sample, triplicate reactions and a control without template were run. PCR conditions followed the procedure in Caporaso *et al*. [27]. Equimolar amplified samples were pooled, cleaned using Qiagen QIAquick PCR Clean Up Kit (Valencia, CA), and sequenced on the MiSeq Illumina platform with a 250bp paired-end approach [26] at the Dana Farber Cancer Institute’s Molecular Biology Core Facilities (Boston, MA). Sequences are available in the NCBI Sequence Read Archive (SRA) under study accession number SRP062876.

Paired reads were assembled with Fastq-join [28] and processed with QIIME (v. 1.7.0) [29]. Sequences were de-multiplexed and quality-filtered (based on [30], with sequences between 252-255bp maintained). After quality filtering, there were 18,299,242 reads (number of sequences per sample: 27,693–176,285). Quality-filtered sequences were clustered into operational taxonomic units (OTUs) at a sequence similarity threshold of 97% with the UCLUST method [31] and a minimum cluster size of 0.001% of the total reads [30]. Sequences were clustered against the 12_10 Greengenes database (October 2012 release) [32], and those that did not match the database were clustered *de novo* at 97% sequence similarity. The most abundant sequence for a given cluster was assigned as the representative sequence for that OTU. Taxonomy was assigned with RDP classifier [33] using the Greengenes database [32]. Representative sequences were aligned to the Greengenes database with PyNAST [34] and a phylogenetic tree was constructed with FastTree [35]. After the 0.001% OTU threshold was implemented, 2023 OTUs remained and the number of sequences per sample ranged from 25,669 to 161,652. All samples were rarefied to 25,000 sequences to standardize sampling effort prior to subsequent analyses.

### Metabolite analysis

To examine skin community function, we characterized the metabolite profiles of individual skin swabs using high performance liquid chromatography/mass spectroscopy (LC/MS) with quadrupole, time-of-flight (qTOF) mass detection and Agilent MassProfiler software. Our method focused on small (<600 m/z), non-polar metabolites, which are likely produced by bacteria as opposed to the host (e.g. anti-microbial peptides). To extract the metabolites, 125μL of HPLC grade methanol was added to each tube containing a swab, vortexed for 3sec and left for 10min. Methanolic extracts were evaporated to dryness *in vacuo*. Crude, recovered material was reconstituted in 50μL of 30% LC/MS grade acetonitrile and 70% LC/MS grade water. The tube was then vortexed for 3sec and left for 5min.

A 6530 Accurate-Mass qTOF mass spectrometer coupled to a 1290 Infinity UHPLC pump (Agilent Technologies, Santa Clara, CA) was used for sample analysis. Each sample was run in triplicate with blank runs between samples. Five μL of sample was injected into the HPLC equipped with an EclipsePlusC18 column (2.1 x 100 mm) that was held at 50C. Compounds were eluted at a flow rate of 0.3 mL/min with mobile phases of water with 0.1% formic acid (A) and acetonitrile with 0.1% formic acid (B). Mobile phase A decreased from 95% to 5% in 4.5min, after which the mobile phase composition returned to the initial conditions and held until 5min. A 1.5min post-run (95% A) ensured the column was flushed and equilibrated for the next injection. After separation, compounds were ionized for mass spectrometric detection by negative ion ESI at -3000V. Agilent MassProfiler software was used to compare the samples to a blank. Compounds found in the blank were subtracted from the bullfrog samples.

The initial metabolite dataset contained 18,603 distinct compounds. To focus on the most dominant metabolites and reduce the high dimensionality of the dataset for analysis, several filtering steps were performed. As a first filter, a metabolite was included if it appeared in all three replicates of a sample and was present on at least five of the 180 samples across all samples and time points. In addition, as our antibiotic treatment was effective at significantly decreasing bacterial diversity at day 0, we used that as a set point for the metabolite analysis, and focused on the metabolites that were absent on all three replicates of antibiotic-treated frogs immediately following antibiotic treatment (day 0). These filtering steps resulted in a final day 7 dataset that consisted of 176 metabolites.

### Bd infection analysis

To quantify Bd infection, we used a quantitative, real-time PCR (qPCR) assay developed by Boyle et al. [36]. Each run included a series of five dilution standards ranging in concentration by an order of magnitude from 0.1 to 1000 zoospore equivalents, as well as a negative control. Bd strain JEL310 was used to create the standards, which were run in triplicate. Samples were initially run in singlicate; however, positive samples were rerun 1–2 more times to confirm the results and more accurately estimate infection intensity. Samples with <0.1 zoospore estimates were considered negative for the presence of Bd because accuracy outside the range of the standards is unknown, and low intensities are especially susceptible to indeterminate results [37]. To confirm that inhibition was not affecting amplification, an internal positive control (Taqman Exogenous Internal Positive Control reagents, Applied Biosystems, Foster City, CA, USA) [38] was added to one of the three triplicate reactions for several randomly selected samples.

### Statistics

We examined the effects of the microbiota manipulations and Bd treatments on Bd infection intensity, microbial community structure (beta and alpha diversity, *J*. *lividum* abundance), and function (frog growth, metabolite profiles). Using individual-based models, we tested for relationships between microbial community structure and Bd infection intensity, microbial community structure and frog growth, Bd infection intensity and frog growth, and microbial community structure and function. Below are the specific statistical methods we used to address each of these relationships.

Bd infection was characterized by prevalence (proportion of individuals infected) and intensity (number of Bd zoospore equivalents). Treatment effects on Bd infection intensity at day 7 were analyzed with a generalized linear model (GLM) using a normal distribution. Bd infection intensity at day 7 was log transformed to obtain a normal distribution. Transformation of Bd infection intensity at day 42 did not result in a normal distribution; therefore, treatment effects on Bd infection intensity at day 42 were analyzed with a Wilcoxon nonparametric ANOVA.

To examine treatment effects on frog growth, proportional growth between the beginning (Day 0) and end (Day 42) of the experiment was calculated and used as the response variable in a mixed model ANOVA with frog as a random effect. Day 0 was used as a baseline in this analysis because there was no difference in mass among the six treatment groups at the start of the experiment (ANOVA: df = 5, F = 0.797, P = 0.557). Contrasts were used to compare growth between unmanipulated and antibiotic- or *J*. *lividum*-treated frogs in different Bd environments. To determine if individuals’ Bd infection intensity predicted frog growth at the end of the experiment, linear regression was used to investigate proportional growth at Day 42 in response to day 7 log-transformed Bd infection intensity.

Differences in microbial community structure (beta diversity of OTUs) across treatments were examined statistically by PERMANOVA on the relative abundance- and phylogenetic-based weighted UniFrac distance matrices. Differences in microbial community function (metabolite profiles) were also analyzed by PERMANOVA on the incidence-based Sorensen dissimilarity matrix generated from day 7 metabolite profiles. Pair-wise tests between treatments were performed on the matrices. For both structure and function matrices, non-metric multidimensional scaling (NMDS) was used to visualize differences among individual frogs. Primer6 v. 6.1.15 and Permanova+ v. 1.0.5 [39] were used for these analyses. To test for a relationship between individuals’ microbial community structure prior to Bd exposure (day 0) and Bd infection intensity one week following exposure (day 7), a Mantel test was performed using the day 0 weighted UniFrac distance matrix and the day 7 Bd infection intensity Euclidean distance matrix. To test for a relationship between individuals’ microbial community structure and function at day 7, one week following Bd exposure, a Mantel test was performed using the day 7 weighted UniFrac distance matrix and the day 7 metabolite Sorensen matrix.

To test for treatment effects (microbiota manipulation, Bd exposure, and their interaction) on alpha diversity (OTU richness and phylogenetic diversity) one week after exposure to Bd, generalized linear models using a normal error distribution were used. Pair-wise contrasts were conducted for each analysis. To determine if individuals’ alpha diversity was correlated with Bd infection intensity or frog growth at the end of the experiment, linear regression was used to evaluate Bd infection intensity at day 7 or proportional growth at day 42 in response to day 7 OTU richness or phylogenetic diversity. The alpha diversity metrics of OTU richness and phylogenetic diversity were computed on rarefied data with MacQIIME.

The relative abundance of *J*. *lividum* OTUs was compared across bacterial treatments using a nonparametric Wilcoxon ANOVA followed by Steel-Dwass multiple comparisons. This was done using the Illumina dataset, with the relative abundances combined for two *J*. *lividum* OTUs that had ≥99.6% sequence similarity to the isolate used in the experiment.

To identify OTUs driving differences in microbial communities over time and across treatments, the relative abundances of each OTU were compared with Kruskal-Wallis tests with Bonferroni corrected p-values. Lastly, to test for a relationship between OTU relative abundance and Bd infection intensity or growth, linear regression was used.

### Ethics Statement

All procedures were approved by Virginia Tech’s Institutional Animal Care and Use Committee (Protocol 08-042-BIOL). All frogs were euthanized in 0.1% buffered MS–222, which is a humane form of euthanasia for amphibians to minimize animal suffering.

## Results

### Efficacy of microbiota treatments

The two weeks of antibiotics effectively reduced bacterial diversity relative to the unmanipulated controls, both in terms of OTU richness (AB mean±SE = 303±8, U mean±SE = 425±20; ANOVA, F = 31.76, P<0.01) and phylogenetic diversity (AB mean±SE = 28±0.6, U mean±SE = 33±1; ANOVA, F = 16.93, P<0.01).

Initial field swabs indicated *J*. *lividum* was present on 55% (28 of 51) of bullfrogs in the field, with a mean relative abundance of 0.02% (SE = 0.005). However, our *J*. *lividum* treatment still did increase *J*. *lividum* relative abundance at day 0 (Wilcoxon Χ^2^ = 36.6, P<0.01); frogs inoculated with *J*. *lividum* had a higher mean relative abundance of *J*. *lividum* (2.7±0.8%) than unmanipulated (0.03±0.02%) or antibiotic-treated (0.04±0.01%) individuals (Steel-Dwass—JL-U: Z = -4.90, P<0.01; JL-AB: Z = 4.93, P<0.01).

### Bd infection intensity

**Fig 2:**
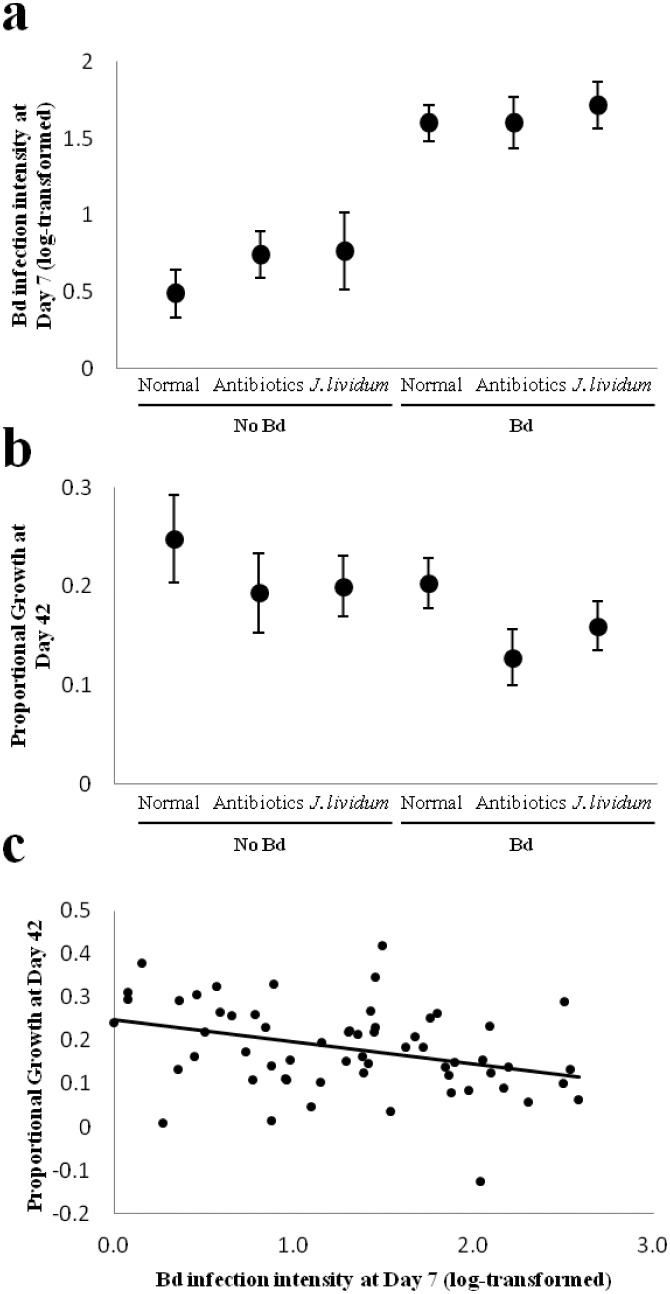
Effects of treatment on bullfrogs’ Bd infection intensity at day 7 (a) and proportional growth at day 42 (b). Error bars represent standard error. The significant relationship between individual bullfrogs’ Bd infection intensity at day 7 and proportional growth at day 42, the end of the experiment (c).

Bd-exposed frogs had significantly higher infection intensity at day 7 than unexposed frogs (Fig 2a; Mean+/-SE zoospore equivalents, Bd: 82.6+/-16, NoBd: 8.9+/-3.6; Bd exposure: Χ^2^ = 37.64, P<0.001), demonstrating successful laboratory infection with Bd. However, manipulating the microbiota did not affect Bd infection intensity, and there was no interaction between Bd exposure and microbiota manipulation on Bd infection intensity at day 7 (Fig 2a; Microbiota manipulation: Χ^2^ = 1.48, P = 0.5; Microbiota manipulation*Bd exposure: Χ^2^ = 0.57, P = 0.8).

### Frog Growth

All frogs survived and gained weight over time, but those exposed to Bd tended to grow less over the course of the experiment than those not exposed to Bd (Fig 2b; F = 3.63, P = 0.06). Microbiota manipulation treatment did not affect growth (F = 2.15, P = 0.1), and there was no interaction between microbiota manipulation and Bd (F = 0.09, P = 0.9). However, when exposed to Bd, antibiotic-treated frogs grew significantly less than frogs with their microbiota unmanipulated (Fig 2b; AB,Bd-U,Bd: t = -1.98, P = 0.05). Without exposure to Bd, there was no difference in growth between unmanipulated and antibiotic-treated frogs (AB,NoBd-U,NoBd: t = -1.05, P = 0.3). Augmentation with *J*. *lividum* did not significantly affect growth either with (JL,Bd-U,Bd: t = -1.14, P = 0.26) or without exposure to Bd (JL,NoBd-U,NoBd: t = 0.13, P = 0.89). There was a significant relationship between individual frogs’ Bd infection intensity at day 7 and their growth at day 42 (F = 8.27, P = 0.006, R^2^ = 0.12), such that frogs with higher infection tended to grow less (Fig 2c).

### Microbial Community Structure—Beta diversity

Bringing bullfrogs into the laboratory changed their microbial communities (U,NoBd frogs only, initial-day0: t = 2.57, P = 0.002), but the communities were relatively stable between days 0 and 7 (U,NoBd frogs only, day0-day7: t = 1.21, P = 0.15). Despite the laboratory housing effect, microbiota manipulation resulted in differences in microbial community structure at day 0, immediately following microbiota manipulation and prior to Bd exposure (PERMANOVA: Pseudo-F = 15.21, P<0.001). Antibiotic treatment (AB-U: t = 4.30, P<0.001) and addition of *J*. *lividum* (JL-U: t = 1.62, P = 0.01) altered the normal microbial community. Different microbial community structures (weighted UniFrac) at day 0 were significantly associated with variation in Bd infection intensity at day 7 (Bd-exposed frogs only, Mantel r statistic = 0.15, P = 0.03).

**Fig 3:**
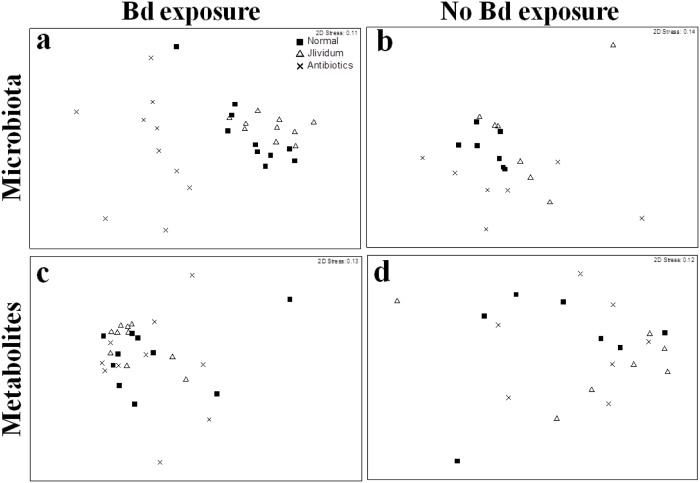
NMDS ordinations based on weighted UniFrac distance matrices (a,b) and Sorensen dissimilarity matrices (c,d) representing differences among microbiota manipulations in microbial community structure and metabolite profiles, respectively, of frogs exposed (a,c) and unexposed (b,d) to Bd one week after initial exposure to Bd. There were differences in microbial community structure and metabolite profiles among microbiota manipulation only with Bd exposure.

Exposure to Bd significantly affected microbial community structure one week post-exposure (day 7; PERMANOVA: Pseudo-F = 3.34, P = 0.008). Interestingly, there was an interaction between microbiota manipulation and Bd exposure treatments, such that *J*. *lividum* and antibiotic treatment significantly altered the normal microbiota only when individuals were also exposed to Bd (Fig 3a and 3b; Table 1; Bd exposed: Pseudo-F = 9.66, P<0.001; Not Bd exposed: Pseudo-F = 1.58, P = 0.06). To determine if particular OTUs were driving these differences in community structure among Bd exposed individuals, we compared relative abundances of each OTU across microbiota manipulation treatments and identified 37 OTUs that differed significantly across microbiota manipulation treatments at day 7, including *Janthinobacterium* OTUs (Table 2; sequence data in S1 Text). Among these 37 OTUs, a Comamonadaceae OTU (Table 2; OTU ID: denovo138967) exhibited the highest overall relative abundance, and was highest in the unmanipulated treatment (20.7% mean relative abundance +/- 3.2% SE), medium in the *J*. *lividum* treatment (5.9% mean relative abundance +/- 2.0% SE), and very low in the antibiotic treatment (0.06% mean relative abundance +/- 0.008% SE). Other significantly different OTUs typically had low mean relative abundance (<0.6%). Additionally, the relative abundance of the Comamonadaceae OTU on Bd exposed frogs at day 7 was positively correlated with day 42 growth, but not Bd infection intensity (growth: F = 5.79, P = 0.02, R^2^ = 0.17; Bd infection intensity: F = 0.21, P = 0.6, R^2^ = 0.007). Among frogs not exposed to Bd, however, relative abundance of the Comamonadaceae OTU was not associated with frog growth (F = 0.016, P = 0.9, R^2^ = 0.0008).

**Table 1:** Summary of PERMANOVA results comparing microbial community structure (beta diversity of OTUs, UniFrac distance matrices) and function (Metabolite profiles, Sorensen dissimilarity matrices) across microbiota manipulations of frogs exposed and not exposed to Bd. Visualizations of these comparisons can be seen in Fig 3.

	OTUs		Metabolites	
Bd Treatment	Pseudo-F	P value	Pseudo-F	P value
Exposed to Bd	9.66	<0.001	1.72	0.01
Not exposed to Bd	1.58	0.06	1.16	0.3

**Table 2:** List of 37 OTUs that differed significantly in relative abundance among microbiota manipulation treatments (antibiotics, augmented with *J*. *lividum*, and unmanipulated) 7 days after exposure to Bd, based on a Kruskal-Wallis test with Bonferroni corrected P values. Taxonomy includes the phylum and the lowest classification that could be defined for each OTU. Phyla are abbreviated as follows: Act = Actinobacteria, Bac = Bacteroidetes, Pro = Proteobacteria. OTUs are ranked in order of relative abundance in the unmanipulated treatment. The Comamonadaceae OTU that was positively correlated with frog growth among Bd-exposed frogs is bolded. Representative 16S rRNA gene sequences for these 37 OTUs can be found in S1 Text.

		Mean relative abundance (%)				
OTU ID	Taxonomy	Antibiotics	*J*. *lividum*	Unmanipulated	Test-Statistic	Bonferroni P
**denovo138967**	**Pro: Comamonadaceae**	**0.06**	**5.89**	**20.68**	**23.29**	**0.02**
denovo7503	Pro: Burkholderiales	0	0.16	0.43	21.47	0.04
denovo15797	Pro: *Acinetobacter*	0	0.11	0.3	23.05	0.02
denovo101948	Pro: Comamonadaceae	0	0.06	0.17	24.78	0.01
denovo26623	Pro: Comamonadaceae	0	0.04	0.12	23.11	0.02
95469	Pro: Burkholderiales	0	0.02	0.11	23.77	0.01
denovo142751	Pro: Comamonadaceae	0	0.04	0.1	23.23	0.02
denovo172928	Pro: *Acinetobacter*	0	0.39	0.09	26.79	0
denovo146080	Pro	0	0.03	0.07	22.41	0.03
denovo20210	Pro: Comamonadaceae	0	0.01	0.06	22.5	0.03
denovo120482	Pro: Comamonadaceae	0	0.01	0.06	22.87	0.02
denovo83658	Pro: Burkholderiales	0	0.01	0.05	21.83	0.04
denovo17986	Pro: Betaproteobacteria	0	0	0.03	21.98	0.03
104023	Pro: *Janthinobacterium*	0	0.62	0.02	24.36	0.01
denovo155383	Pro: *Pseudomonas*	0	0	0.02	22.26	0.03
denovo165786	Pro: Burkholderiales	0	0.32	0.01	23.93	0.01
denovo60162	Pro: *Janthinobacterium*	0	0.16	0.01	24.54	0.01
denovo136414	Pro: Moraxellaceae	0	0.02	0.01	22.31	0.03
denovo73976	Pro: Comamonadaceae	0	0	0.01	21.9	0.04
4425629	Pro: Oxalobacteraceae	0	0.2	0.01	22.89	0.02
denovo76188	Pro: Betaproteobacteria	0	0.03	0	22.81	0.02
denovo2839	Pro: *Janthinobacterium*	0	0.1	0	24.33	0.01
denovo140152	Pro: Burkholderiales	0	0.03	0	22.93	0.02
denovo34254	Pro: Burkholderiales	0	0.03	0	22.69	0.02
4393701	Pro: *Janthinobacterium*	0	0.05	0	23.78	0.01
denovo117711	Pro: Betaproteobacteria	0	0.07	0	26.05	0
2279387	Pro: *Janthinobacterium*	0	0.02	0	25.19	0.01
denovo101210	Pro: Betaproteobacteria	0	0.01	0	25.11	0.01
denovo106079	Pro: Burkholderiales	0	0.01	0	22.5	0.03
denovo143492	Act: Actinomycetales	0.08	0	0	25.03	0.01
denovo134685	Pro: Burkholderiales	0	0.02	0	26.11	0
denovo95632	Pro	0.08	0	0	25.78	0.01
denovo47581	Bac: *Chryseobacterium*	0.02	0	0	21.69	0.04
denovo76057	Pro: Pseudomonadaceae	0	0.02	0	23.89	0.01
denovo43925	Pro: Comamonadaceae	0.06	0	0	22.13	0.03
denovo145740	Pro: Xanthomonadaceae	0.05	0	0	23.88	0.01
denovo163457	Bac: *Chryseobacterium*	0.01	0	0	23.94	0.01

### Microbial Community Function—Metabolite Profiles

Similar to the effects of Bd on microbial community structure, the metabolite profiles of Bd exposed frogs were significantly different than the profiles of unexposed frogs at day 7 (PERMANOVA: Pseudo-F = 2.78, P<0.01). Also consistent with the community structure data, there was an interaction between microbiota manipulation and Bd exposure treatments, such that microbiota manipulation affected metabolite profiles only on individuals also exposed to Bd (Fig 3c and 3d; Table 1; Bd exposure: Pseudo-F = 1.72, P = 0.01; No Bd exposure: Pseudo-F = 1.16, P = 0.3). Furthermore, day 7 microbial community structure correlated with day 7 metabolite profiles (Mantel r statistic = 0.15, P = 0.02).

### Microbial Community Structure—Alpha Diversity

**Fig 4:**
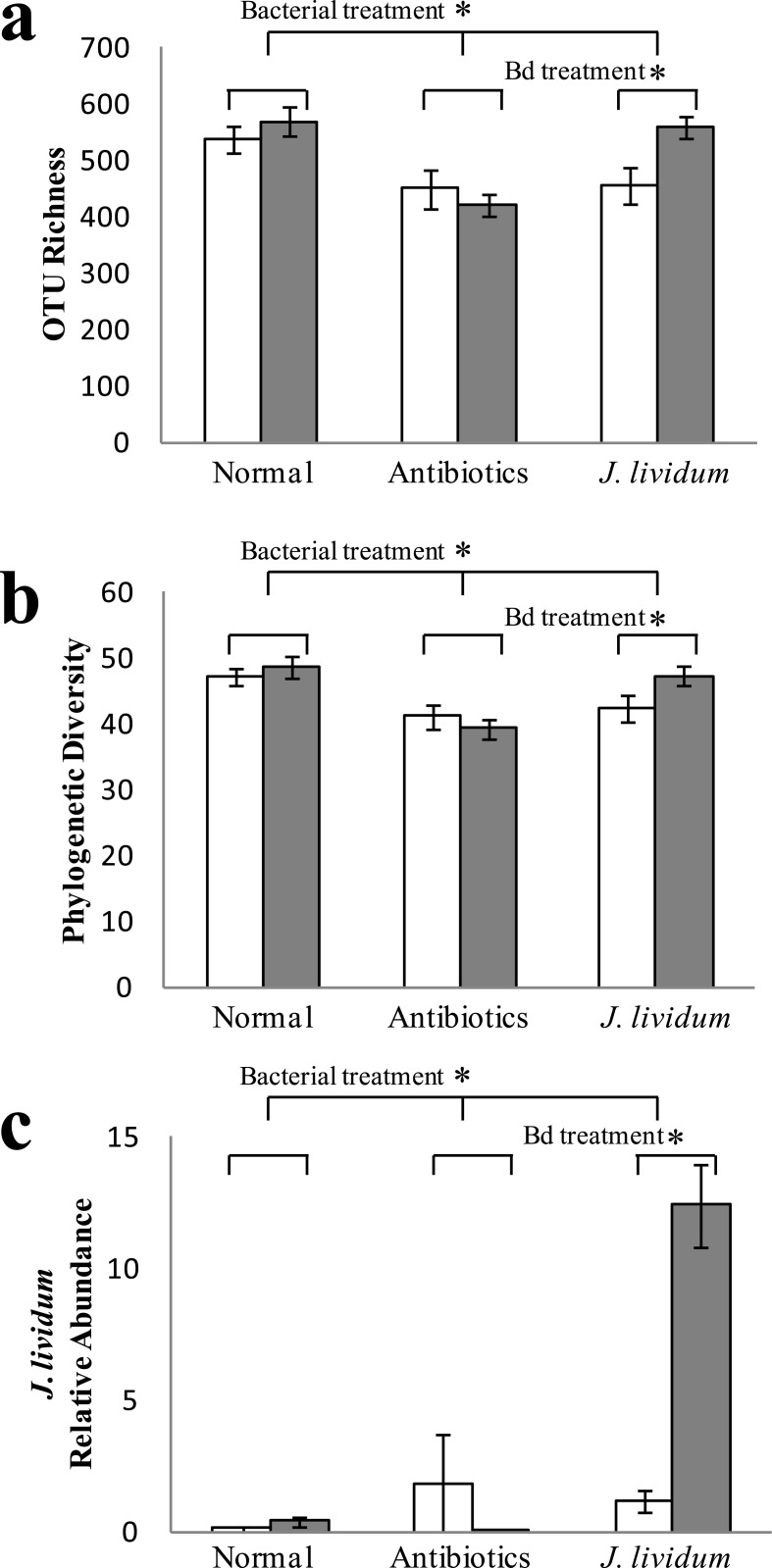
Effects of Bd exposure (white bars = No exposure to Bd; grey bars = Exposure to Bd) and bacterial treatment (normal, antibiotics, and augmented with *J*. *lividum*) on OTU richness (a), phylogenetic diversity (b), and relative abundance of the probiotic *J*. *lividum* (c) on bullfrog skin one week following exposure to Bd (day 7). Error bars represent standard error. * represent significant differences among treatments.

One week after exposure to Bd (day 7), OTU richness was significantly affected by microbiota manipulation, and by an interaction between microbiota manipulation and Bd exposure; however, overall exposure to Bd did not significantly affect OTU richness (Fig 4a; GLM—Microbiota manipulation: Χ^2^ = 18.52, df = 2, P<0.01; Bd exposure: Χ^2^ = 2.76, df = 1, P = 0.1; Microbiota manipulation*Bd exposure: Χ^2^ = 6.33, df = 2, P = 0.04). Frogs treated with antibiotics continued to harbor fewer OTUs than unmanipulated frogs (Χ^2^ = 18.25, P<0.01). Interestingly, *J*. *lividum*-treated frogs that were exposed to Bd had greater OTU richness than those not exposed to Bd (Fig 4a; Χ^2^ = 7.65, P<0.01). However, within the unmanipulated or antibiotic treatments, there was no effect of Bd on OTU richness (U,Bd-U,NoBd: Χ^2^ = 0.69, P = 0.4; AB,Bd-AB,NoBd: Χ^2^ = 0.62, P = 0.4). Phylogenetic diversity at day 7 was significantly affected by microbiota manipulation, but not Bd treatment or their interaction (Fig 4b; GLM—Microbiota manipulation: Χ^2^ = 18.5, P<0.01; Bd exposure: Χ^2^ = 1.23, P = 0.3; Microbiota manipulation*Bd exposure: Χ^2^ = 4.02, P = 0.1). Frogs treated with antibiotics had lower phylogenetic diversity than unmanipulated frogs (Χ^2^ = 18.29, P<0.01), and *J*. *lividum*-treated frogs that were exposed to Bd had greater phylogenetic diversity than those not exposed to Bd (Fig 4b; Χ^2^ = 4.18, P = 0.04).

**Fig 5:**
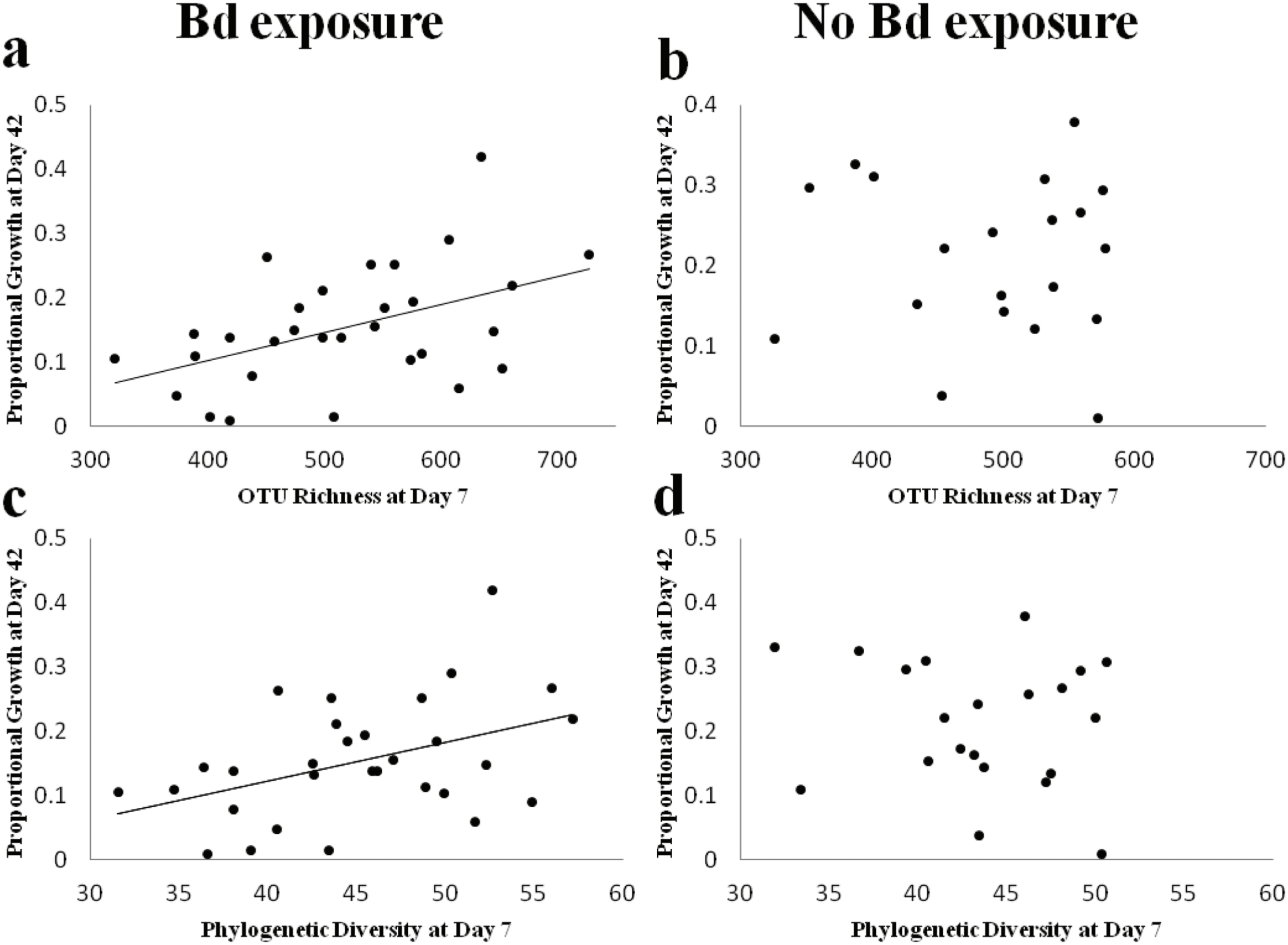
Correlations between OTU richness (a,b) or phylogenetic diversity (c,d) at day 7 and proportional growth at day 42, with (a,c) and without (b,d) exposure to Bd.

OTU richness and phylogenetic diversity at day 7 were, in turn, positively correlated with growth of Bd exposed frogs (Fig 5a and 5c; OTU richness: F = 8.11, P<0.01; Phylogenetic diversity: F = 6.38, P = 0.02), but there was no correlation between diversity and growth of unexposed frogs (Fig 5b and 5d; OTU richness: F = 0.59, P = 0.45; Phylogenetic diversity: F = 0.44, P = 0.52). Diversity of bacteria prior to Bd exposure on day 0, however, did not predict growth (day 42) or Bd infection intensity (day 7) regardless of Bd exposure (all tests P>0.05).

### Microbial Community Structure—*J*. *lividum* relative abundance

At day 7, *J*. *lividum*-treated frogs exposed to Bd had higher levels of *J*. *lividum* than unexposed frogs (Fig 4c; Wilcoxon: Χ^2^ = 30.5, df = 5, P<0.01; Steel-Dwass—JL,Bd-JL,NoBd: Z = -3.37, P = 0.01), while Bd exposure did not significantly affect *J*. *lividum* abundance within the antibiotic or unmanipulated treatments (Steel-Dwass—AB,Bd-AB,NoBd: Z = -0.82, P = 0.96; U,Bd-U,NoBd: Z = 0.59, P = 0.99). Providing further support for an interaction between Bd and *J*. *lividum*, within the Bd exposed group, *J*. *lividum*-treated individuals had significantly higher relative abundance of *J*. *lividum* than individuals with an unmanipulated microbiota (Steel-Dwass—JL,Bd-U,Bd: Z = -3.76, P<0.01); however, without exposure to Bd, *J*. *lividum* relative abundance did not differ between *J*. *lividum* and control treatments (Steel-Dwass—JL,NoBd-U,NoBd: Z = -2.63, P = 0.09).

## Discussion

We have experimentally demonstrated that Bd is likely a selective force on amphibian skin bacterial community structure and function. There were differences in community structure and function between unmanipulated, antibiotic-treated, and *J*. *lividum*-treated frogs only when individuals were exposed to Bd. Without the pressure of the pathogen, there were no significant differences in microbial community structure or function among microbiota manipulation treatments. These differences in microbial community structure and function may explain reduced growth of antibiotic-treated frogs only when they were exposed to Bd. Jani and Briggs [40] also recently demonstrated that Bd can alter the composition of the skin microbiome of *Rana sierrae* in both field and laboratory settings. However, Becker et al. [41] found that Bd exposure did not affect the skin microbiome of *Atelopus zeteki*. In our study, we found that Bd can affect the structure and function of bullfrogs’ skin microbiome, but also that the microbiome can affect Bd infection. As noted by Jani and Briggs [40], these responses (“Bd-induced disturbance” and “bacteria-induced resistance”) are not mutually exclusive.

We found that microbial community structure prior to Bd exposure was correlated with Bd infection intensity one week post Bd exposure. One possible explanation for this is that bacterial symbiont communities can limit infection, but that the extent of that defense is likely dependent on community structure, and subsequent function (metabolite production). Another recent study found similar associations with skin microbial communities in a species that, unlike bullfrogs, is highly susceptible to Bd; *Atelopus zeteki* survival following exposure to Bd was correlated with the initial composition of the skin bacterial communities [41]. This relationship between host-associated microbiota and disease outcomes has been observed in other systems as well (e.g. [42–44]).

The correlation between initial microbial community structure and Bd infection intensity could also be explained by factors that drive microbial community structure and are also important in response to Bd. For example, other aspects of the immune system, such as antimicrobial peptides, [45–47] or host genetics [48,49] may be the underlying mechanism of this association. Nonetheless, knowing whether a disease-limiting microbial community structure exists can inform efforts to manipulate the microbiome to increase host defenses. For instance, root exudates of plants can be used to modify the rhizosphere microbiome to promote disease suppression [50], and in humans, entire fecal microbiota transplants can eliminate recurrent *Clostridium difficile* infections [51].

We identified a direct link between community structure and function, and both were altered by pathogen exposure. Therefore, according to our conceptual model (Fig 1), we hypothesize that one or more key bacteria have a role in driving these patterns, an observation that has also been made in aquatic microbial communities, where certain metabolic functions are mediated by individual phylotypes, as opposed to being related to community diversity [52]. Within Bd exposed frogs, we identified 37 OTUs that differed significantly in mean relative abundance across microbiota manipulation treatments. One of these OTUs, in the family Comamonadaceae, may be a key member in these communities, as its relative abundance at day 7 was significantly correlated with growth of Bd exposed frogs. We think selection for this OTU is occurring as a response to Bd exposure because: (1) frog growth was associated with the post-Bd exposure relative abundance of this OTU, but not with the relative abundance prior to Bd exposure, and (2) this relationship existed only among Bd exposed frogs. Members of the Comamonadaceae family are abundant on bullfrogs and other amphibians, and some cultured isolates from this family can inhibit Bd growth *in vitro* [12,13,53]. It is therefore plausible that this bacterial group may play a key role in limiting Bd infection for amphibian hosts. Because we did not isolate the OTUs or metabolites characterized in these profiles and test them for inhibitory capabilities against Bd directly, the differences in structure and function observed is merely suggestive of selection on the function of the microbial communities in response to Bd. Additional tests of Bd inhibition by specific OTU and metabolite profiles would fully test this hypothesis.

Communities with high diversity are often considered more resistant to invasion than communities with low diversity, although there is some debate about this relationship [54,55]. In diverse microbial systems, including wheat rhizospheres, locust guts, and soil microcosms, species-rich microbial communities are more resistant to pathogen invasion than species-poor microbial communities [56–58]. In our study, there was no correlation between alpha diversity *per se* (OTU richness and phylogenetic diversity) and Bd infection intensity. However, among Bd exposed frogs, diversity *per se* was positively associated with host growth. This suggests that more diverse microbial communities may not always limit pathogen growth directly, but may still reduce host morbidity, and potentially mortality, following pathogen exposure.

In our experiment, microbial community structure appeared to respond to pathogen exposure in a context-specific manner. For instance, within the *J*. *lividum* treatment, Bd exposed frogs had higher OTU richness, phylogenetic diversity, and *J*. *lividum* relative abundance than non-exposed frogs. This suggests an interaction between Bd and *J*. *lividum*, such that high *J*. *lividum* abundance and overall diversity is maintained by pathogen exposure. Jani and Briggs [40] also found that a *Janthinobacterium* OTU was more abundant on frogs experimentally infected with Bd than on uninfected frogs. Interestingly, Bd metabolites can enhance the growth of *J*. *lividum in vitro* [59]. The maintenance of *J*. *lividum* in the skin bacterial community and its effectiveness against Bd may, in fact, be facilitated by Bd.

We hypothesized that a microbiota augmented with the anti-Bd bacterium *J*. *lividum* would decrease susceptibility of bullfrogs to Bd by decreasing infection intensity. However, there were no differences in Bd infection intensity among *J*. *lividum*-treated frogs and frogs with an unmanipulated microbiota that were exposed to Bd. This result was also observed in *A*. *zeteki*, where there were no differences in Bd loads between Bd only and Bd plus *J*. *lividum* treatments [60]. In addition, we thought that a reduced microbiota might increase susceptibility of bullfrogs to Bd. While antibiotic-treated frogs did not have significantly higher Bd infection intensities, they grew less than frogs with unmanipulated microbiota following Bd exposure. At the individual level, the day 0 microbial community structure of bullfrogs predicted Bd infection intensity at day 7, which then predicted growth at day 42. Thus, the normal microbiota of bullfrogs is important for disease outcome, and potentially host fitness. Results of other amphibian studies also suggest that the skin microbiota plays a role in host response to Bd infection [15,61]. Therefore, factors that contribute to microbial community assembly and maintenance on amphibian skin, including host factors [62], habitat [63], diet [64], and the available microbial species pool [12,65], may ultimately influence disease dynamics.

Taken together, we found that microbial community structure influenced disease-limiting function in amphibians. Studies such as this, that experimentally manipulate complex host-associated microbial communities and potential selective forces, are valuable for understanding structure-function patterns and regulating mechanisms in diverse systems [8].

## Supporting Information

S1 Text Representative 16S rRNA gene sequences for 37 OTUs that differed significantly in relative abundance among microbiota manipulation treatments (antibiotics, augmented with *J*. *lividum*, and unmanipulated) 7 days after exposure to Bd. The representative sequence was defined as the sequence that was most abundant in the OTU cluster.
(TXT)
